# Genetic structure of *Trypanosoma cruzi* in Colombia revealed by a High-throughput Nuclear Multilocus Sequence Typing (nMLST) approach

**DOI:** 10.1186/1471-2156-14-96

**Published:** 2013-09-30

**Authors:** Juan David Ramírez, Gabriela Tapia-Calle, Felipe Guhl

**Affiliations:** 1 Centro de Investigaciones en Microbiología y Parasitología Tropical (CIMPAT), Universidad de Los Andes, Bogotá, Colombia

**Keywords:** Chagas disease, Clonality, Sexuality, Disease ecology, Transmission dynamics, Genotypes

## Abstract

**Background:**

Chagas disease is a systemic pathology caused by *Trypanosoma cruzi*. This parasite reveals remarkable genetic variability, evinced in six Discrete Typing Units (DTUs) named from *T. cruzi* I to *T. cruzi* VI (TcI to TcVI). Recently newly identified genotypes have emerged such as TcBat in Brazil, Colombia and Panama associated to anthropogenic bats. The genotype with the broadest geographical distribution is TcI, which has recently been associated to severe cardiomyopathies in Argentina and Colombia. Therefore, new studies unraveling the genetic structure and natural history of this DTU must be pursued.

**Results:**

We conducted a spatial and temporal analysis on 50 biological clones of *T. cruzi* I (TcI) isolated from humans with different clinical phenotypes, triatomine bugs and mammal reservoirs across three endemic regions for Chagas disease in Colombia. These clones were submitted to a nuclear Multilocus Sequence Typing (nMLST) analysis in order to elucidate its genetic diversity and clustering. After analyzing 13 nuclear housekeeping genes and obtaining a 5821 bp length alignment, we detected two robust genotypes within TcI henceforth named TcI_DOM_ (associated to human infections) and a second cluster associated to peridomestic and sylvatic populations. Additionaly, we detected putative events of recombination and an intriguing lack of linkage disequilibrium.

**Conclusions:**

These findings reinforce the emergence of an enigmatic domestic *T. cruzi* genotype (TcI_DOM_), and demonstrates the high frequency of recombination at nuclear level across natural populations of *T. cruzi*. Therefore, the need to pursue studies focused on the diferential virulence profiles of TcI strains. The biological and epidemiological implications of these findings are herein discussed.

## Background

Parasitic diseases represent one of the main problems in public health systems, especially in developing countries. The studies focused on determining the genetic diversity of parasites is mandatory, as well as the study of the propagation mechanisms displayed by these microorganisms. These mechanisms have been partially elucidated in *Toxoplasma gondii, Trypanosoma brucei, Trypanosoma cruzi, Leishmania, Giardia* and other parasitic protozoa [1-5]. The preponderant clonal evolution (PCE), cryptic sexuality, and mixed of both mechanisms are explanatory of the propagation alternatives of most parasitic protozoa. Despite of intensive effort of different researchers, these theories still remain under intensive debate. Hence, understanding the propagation method employed by the parasitic protozoa may have important implications in the disease prevalence, which suggest a potential topic for the synergy between population genetics and public health systems.

Chagas disease is a zoonosis caused by *Trypanosoma cruzi*, affecting over 10 million people in endemic areas and has recently gained importance due to the cases of immigrant Chagas disease patients in Spain, USA, Switzerland, Canada and other non-endemic countries [6]. The kinetoplastid *T. cruzi* displays a remarkable genetic variability evidenced in at least six Discrete Typing Units (DTUs) broadly distributed in the American continent [7-9]. Within these DTUs, there are hybrid groups as a consequence of genetic exchange. In the history of this parasite, TcI and TcII emerge as the natural ancestors followed by recombination events that originated TcIII and TcIV known as homozygous hybrids [10-12]. The DTUs TcV and TcVI are associated to the domestic cycle of transmission and considered heterozygous hybrids [13].

*Trypanosoma cruzi* I (TcI) is considered the DTU with the broadest geographical distribution and overlaps the domestic and sylvatic cycles of transmission [8]. The ubiquitous distribution of this genotype and its presence in different hosts (humans, mammal reservoirs and triatomines) allows it to display different methods of diversifying selection as previously reported [14]. Many attempts have been carried out to elucidate the intraspecific genetic variability within TcI where a variety of molecular markers have been applied for this purpose (single-locus, mitochondrial multilocus sequencing and multilocus microsatellite strategies) reporting genotypes associated to the domestic, peridomestic and sylvatic cycles of transmission [5,15-19]. Most of these reports suggest events of cryptic substructuring [20]. The evidence of newly described genotypes and subdivision within TcI has been corroborated by reports of maxicircle mosaic recombinants within natural populations in Colombia including patterns of super-infection/co-infection [19].

Multilocus Sequence Typing (MLST) has recently been applied to a wide number of pathogens and emerges as a potential molecular tool to unravel the genetic structure of microorganisms [21,22]. This technique allows the determination of multilocus allelic profiles from individual samples that can be further analyzed by distinct evolutionary and genetic methods. MLST schemes have been developed in *T. cruzi* reporting more than 10 gene fragments as potential markers for DTU discrimination [9,23]. These gene fragments have been useful to establish the genetic structure of circulating parasites in the Argentinean Chaco region including the plausible signals of recombination in different datasets of reference strains [9]. A few number of studies have so far implemented MLST schemes to study the diversity at intra-DTU level. A recent approach has used mitochondrial MLST schemes to examine genetic diversity in biological clones suggesting introgression and cryptic sexuality [19,24]. Despite of these efforts, the rate of evolution of mitochondrial DNA is much faster than nuclear gene fragments, suggesting that MLST schemes based on nuclear data must be conducted. This premise invoked aiming this work to test a selected set of nuclear MLST markers to unravel the genetic structure of TcI in Colombia, with special emphasis on recombination, linkage disequilibrium, cryptic subdivision and evolutionary trends in order to establish a more accurate picture of TcI in Colombia.

## Results

### Genetic diversity and phylogenetic reconstructions

A total of 5821 bp length alignment was analyzed when the 13 gene fragment regions were concatenated. The genetic diversity calculations showed a high degree of intraspecific variation at intra-DTU level (Table 1). The calculations allowed determining a total of 254 polymorphic sites (4%). In this sense, six genes were highly polymorphic (GPX, PDH, GTP, RHO1, SODA and LYT1). Additionally, the genotypic diversity and the number of genotypes showed 191 distinct genotypes across the dataset. The π and θ nucleotide diversity indexes were on average 0.01517 and 0.02821 respectively. We also calculated the typing efficiency according to the nMLST scheme observing the highest number of different genotypes per polymorphic sites for HMCOAR, PDH, RHO1, LAP, GPI, LYT1, STPP2, RB19 and TR; and the lowest values for GPX, GTP, SODA and SODB.

**Table 1:** Parameters of genetic diversity of the 13 nuclear regions using a nMLST approach

**Gene fragment**	**π**^**a**^	**θ**^**b**^	**Number of polymorphic sites**	**Genotypic diversity**	**Number of genotypes**	**Typing efficiency**^**c**^	**Ratio of NS to SN changes**^**d**^
GPX	0.0897	0.20069	35	0.959	29	0.087	0.654
HMCOAR	0.00515	0.00828	16	0.792	11	0.736	0.659
PDH	0.00467	0.00713	20	0.766	14	0.833	0.127
GTP	0.02268	0.06233	38	0.941	24	0.163	0.223
STPP2	0.00439	0.00581	14	0.848	13	0.500	1.564
RHO1	0.01457	0.01656	26	0.959	28	0.771	1.759
SODA	0.00704	0.00979	19	0.893	14	0.377	0.427
SODB	0.01299	0.02177	28	0.763	8	0.208	0.369
LAP	0.00231	0.00290	7	0.424	6	0.857	0.0125
GPI	0.00001	0.00145	3	0.212	2	0.666	0.0698
LYT1	0.01722	0.02261	27	0.966	28	1.037	0.831
RB19	0.00873	0.00377	11	0.401	7	0.636	0.507
TR	0.00772	0.00357	10	0.383	7	0.700	1.352

^a^π is an index of nucleotide diversity. This measure is defined as the average number of nucleotide differences per site between any two DNA sequences chosen randomly from the sample population.

^b^The mutation parameter (**θ**) is defined as 4 Nm for autosomal loci of diploid organisms, where N is the effective population size (diploid individuals) and m is the neutral mutation rate (per gene or per base pair) per generation.

^c^Typing efficiency is defined as the number of different genotypes described per polymorphic site.

^d^SNPs in the coding region of a gene are of two types, synonymous (SN) and nonsynonymous (NS) SNPs. Synonymous SNPs do not affect the protein sequence while nonsynonymous SNPs change the amino acid sequence of protein.

**Figure 1:**
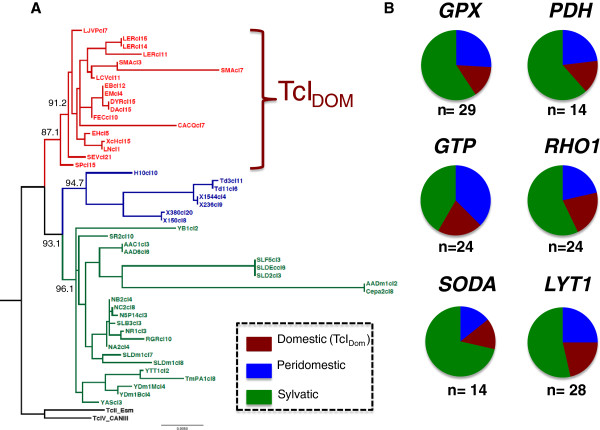
**ML phylogenetic reconstruction and genotype distribution. A**. Phylogenetic reconstruction using the 13 nuclear genetic markers concatenated demonstrating two robust clades within TcI biological clones (in red: domestic isolates; in blue = peridomestic isolates; in green = sylvatic isolates). Genotype distribution. **B**. The piecharts demonstrate the genotype distribution according to six polymorphic genes of TcI_DOM_ and peridomestic/sylvatic clones.

Robust ML phylogenetic trees were constructed for the 13 gene fragments independently supported by iterated bootstrapping analyses. Incongruence among independent tree topologies was observed in six of the gene fragments evaluated (PDH, RHO1, LAP, SODB, LYT1 and GTP) (Additional file 1: Figure S1). The whole dataset was concatenated obtaining 5821 bp SNPs enough for discrimination of robust genotypes. In this final ML tree two robust genotypes validated with bootstrap values above 90% were observed, the previously reported TcI_DOM_ genotype associated to human infections and a second genotype associated to peridomestic and sylvatic isolates. The genotype that clustered sylvatic and peridomestic isolates is highly polymorphic including a noticeable difference in the number of genotypes (Figure 1).

### Diploid sequence types (DSTs) analyses

We identified 50 different DSTs (Diploid Sequence Type) from the 50 biological clones studied (Additional file 2: Table S2). These DST data was employed to test evolutionary hypotheses such as the linkage disequilibrium and the occurrence of founders of the genotypes herein described. When the eBURST analysis was interpreted, we detected the plausible presence of two isolates as possible founders of TcI genotypes. Clones TmPA1cl6 and YAScl2 showed to be the founders in the diagram provided by eBURST software. A second set of founders was determined, being N5P14cl3 and LERcl14 giving rise to the TcI_DOM_ isolates. Likewise, when the LIAN software was employed to test the linkage disequilibrium among the dataset, we observed that the multilocus DST analyses support that TcI clones population is in linkage equilibrium when the concatenated dataset was employed (p = 0.0004; Vd = 12.1; Ve = 11.7) and the association index hypothesis was also statistically significant (p = 0.0137). Lastly, using the DST profiles of the TcI biological clones analyzed, NJ trees were constructed and compared with the topology of ML trees when DNA sequences were used. We observed congruence between the robust genotype detection showing the occurrence of TcI_DOM_ and peridomestic/sylvatic clones cluster as demonstrated below (data not shown).

### Amino acid changes and recombination signals

**Figure 2:**
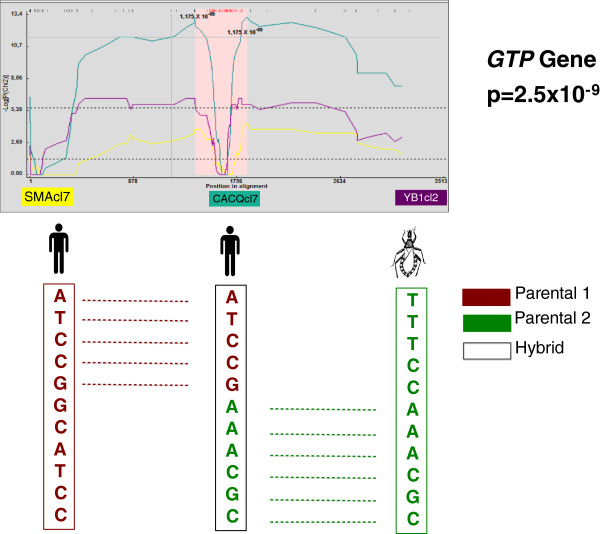
**Recombination detection point output.** Recombination signals in the output of RDP software suggesting frequent events of recombination at nuclear level. Allelic profiles suggest the occurrence of allelic recombination across GTP locus.

The ratios of nonsynonymous (NS) to synonymous (SN) changes were calculated (Table 1). Ten gene fragments showed to be under stabilizing selection showing dN/dS < 1 (GPX, HCOAR, PDH, GTP, SODA, SODB, LAP, GPI, LYT1 and RB19). On the other hand, three single gene fragments were under positive selection showing dN/dS > 1 (STTP2, RHO1 and TR). The ratio was calculated for each gene fragment observing a value on average of 0.359 for the 13 fragments analyzed demonstrating that the majority of genes were under stabilizing selection. Additionally, the 13 gene fragments were concatenated and submitted to analyses on RDP v3.0 in order to check likely events of recombination. We only detected recombinant signals when the MAXCHI algorithm was employed; in total, three robust recombinants were detected (Figure 2). The clone CACQcl7 (p = 2.5 × 10^-9^) showed recombination signals with minor and major parents being SMAcl7 and YB1cl2 respectively. The clone LERcl14 (p = 2.5 × 10^-9^) showed recombination signals with minor and major parents being SMAcl7 and YB1cl2 respectively and the clone SMAcl8 (p = 5.6 × 10^-9^) showed recombination signals with minor and major parents being NA2cl4 and SMAcl3 respectively (Additional file 3: Figure S2). These clones were re-sequenced and cloned in order to check any taq-polymerase slippage obtaining the same sequence pattern.

## Discussion

Multilocus Sequence Typing schemes have proved to be a valuable tool in determining the genetic structure of pathogens and detection of inter-specific diversity [21]. Even though, these approaches have been originally described for bacterial species, new MLST schemes are being deployed for eukaryotes such as *Candida, Batrachochytrium dendrobatidis, Fusarium solani* and *Leishmania*[25-28]. MLST schemes have been useful in order to resolve biological questions such as genetic structure, evolution of reproduction, recombination signals and evolutionary trends of pathogens [19,29,30]. Regarding the *T. cruzi* taxon, two different schemes have been developed for the discrimination of the six reported variants [9,23]. Nevertheless, to date no robust epidemiological studies have emerged using these methodologies. Herein, we presented the first molecular epidemiology survey using nuclear MLST scheme in order to establish genetic structure and diversity at intra-lineage level (TcI).

The results of the genetic diversity parameters calculated, demonstrated tremendous variability among the 13 loci tested (Table 1). On average 4% of the full concatenated sequences (more than 5 kb) were catalogued as polymorphic sites and more than 150 different genotypes were found. This corroborates the divergent pattern displayed within this DTU. This is not novel, since using single loci such as spliced-leader intergenic miniexon gene, cytochrome b and multilocus strategies as microsatellites and mtMLST schemes have demonstrated a high degree of polymorphisms within TcI populations [15,17,18,24]. Despite of observing a marked degree of polymorphisms, there are certain genes that were considered as non-variable such as RB19, GPI, LAP and TR. These genes are clearly conserved across the six DTUs [13,31]. This could explain the lack of resolution when applied to our dataset. Likewise, the values of nucleotide diversity confirms that current nomenclature of the *T. cruzi* taxon is not out of order (on average 0.01517); however, at intra-lineage level this value is elevated which suggest the absence of stability at lineage level and not in accordance with the clonal propagation theory (PCE).

Reliable phylogenetic reconstruction of independent and concatenated gene fragments was carried out (Additional file 1: Figure S1; Figure 1). Most of the tree topologies suggest two clear monophyletic clades with robust bootstrap values. The clones associated to the domestic cycle of transmission (TcI_DOM_) and the clones associated to the peridomestic/sylvatic cycle of transmission. The trees were compared with the NJ trees obtained using the DSTs observing a total congruence among phylogenetic reconstruction using DNA alignments and cladistics clustering. The intriguing observation was the clear incongruence detected among the topologies of the single gene fragments. This is the case for GPX, GTP, STPP2, RB19, GPI, HMCOAR and TR vs. PDH, RHO1, LAP, SODB, LYT1 and GTP where the topologies do not coincide (Additional file 1: Figure S1). This incongruence at nuclear level may suggest recombination and/or chromosomal rearrangements among the dataset. This event of phylogeny incongruence has been reported in other organisms such as *Leishmania, Schistosoma* and *Giardia duodenalis* where genetic exchange has been suggested [32-34]. Despite of this pattern, many authors imply that *T. cruzi* displays clonal propagation with rare recombination events [35]. This phylogeny incongruence may evidence recombination at a high rate among Colombian TcI populations; the recombination at intra-TcI level has already been reported in Ecuadorian populations and at mitochondrial level in Colombia where introgression has also been described [5,19]. In addition, the chromosomal rearrangement not attributed to recombination but attributed to genomic reassortment is not a point to exclude. Recently Lima et al. have reported inter- and intra-strain karyotype heterogeneities suggesting that chromosomal rearrangements have occurred during the evolution of *T. cruzi*[36,37]. When the genes that showed different topology were analyzed (GPX, GTP, GPI and TR), it was not rare to observe they may be under chromosomal rearrangements since they are located in four independent chromosomes (35, 12, 6 and 37) respectively [31].

The DSTs were employed to test evolutionary hypotheses such as linkage disequilibrium and possible founders of specific genotypes using LIAN and eBURST software. The results showed that population is at linkage equilibrium and the values of the association index support the idea of recombination. Likewise, this rate of recombination is supported upon the results provided by eBURST software. The linkage equilibrium is the best-fit measure of recombination among a dataset and widely used specifically in organisms where clonal propagation has been evinced [38-40]. Hence, the significance in the absence of linkage disequilibrium allows us to state that genetic exchange is a frequent mode of propagation among Colombian *T. cruzi* I natural populations. The foreseen absence of preponderant clonality has been reported at nuclear level using microsatellites in this dataset. Likewise, we observed a lack of congruence between nuclear and mitochondrial phylogenies (data not shown) demonstrating for the first time using mtMLST and nMLST schemes the presence of cryptic sexuality within *T. cruzi*[19]*.* Additionally, the number of DSTs obtained was equal to the number of clones analyzed demonstrating the absence of lineage stability in space and time that is not in accordance with the PCE model.

For some authors, the evidence of recombination cannot be pointed out just by detecting incongruence among phylogenies regardless of using nuclear and/or mitochondrial DNA [41]. Nevertheless to deal with this issue we submitted the whole alignment (> 5 kb of nuclear DNA) for the detection of recombination signals using RDP. We detected recombinants using the MAXCHI algorithm finding three robust clones with strong signals of recombination (Figure 2). In all the recombinants, we were able to distinguish minor and major parents among the breaking points. Curiously, the breaking point for the three recombinants was detected in the GTP gene. The three recombinants were isolated from human infections in acute phase and among the parents, there is always one clone belonging to the sylvatic cycle of transmission (isolated from *Rhodnius prolixus*). This finding is paradoxically interesting in terms of detecting emerging genotypes within this DTU and also the enigmatic question regarding the place across the life cycle of *T. cruzi* where recombination occurs. These recombinants and their major and minor parents coincide with the finding of DSTs using eBURST. In this case, two events of recombination emerged obtaining a first ancestry recombination event between one clone from *Triatoma maculata* (TmPA1cl6) and one clone from *Alouatta seniculus* (YAS1cl2); both from an arboreal niche in the sylvatic cycle of transmission of Chagas disease. Moreover, the second event is figured out by one clone from *R. prolixus* (N5P14cl3) and one from human oral infection (LERcl14) giving rise to the specific genotype TcI_DOM._ This is of paramount importance because the recombination signals are always reported in at least one isolate from triatomine and might be implying that recombination may be taking place in the reduviidae insects and not within mammal reservoirs and/or hosts. Insect vectors have played a remarkable role in the detection of recombination in different parasitic protozoa; in this sense, recombination signals have been detected *in vitro* in *Phlebotomus* for the case of *Leishmania donovani* and *Glossina* for the case of *T. brucei* respectively [40,42]. The recombination among parasitic protozoa plays an important role in the diversification of these microorganisms and has been detected *in vitro* and *in vivo*; and needs to be related to the severity of parasitic diseases*.*

The phylogenetic reconstruction using the DNA alignments and DSTs profiles based on the nMLST scheme allowed us to establish two robust monophyletic clades named TcI_DOM_ and one associated to peridomestic/sylvatic clones. This grouping has been established since 2007 when the first subdivision based on spliced leader of the mini-exon gene was conducted [15]. Other researchers continued these efforts but most of the associations were made upon the cycles of transmission within TcI [18,43-45]. This subdivision may not be absolute because in some cases domiciliated insect vectors may cluster with sylvatic population that is not unlikely since they may carry populations of the sylvatic foci as natural predictors of parasite transmission dynamics. In this case, it is more accurate to name the genotypes associated to human infection as an emergent genotype. Robust coalescent Bayesian dating suggest that this genotype emerged approximately 23 000 ± 12 000 years ago and followed by population expansion, broadly corresponding with the earliest human migration into the Americas [19]. Emergent genotypes have been reported in other parasites such as *Plasmodium* and *Leishmania*[46-48] as a strong signal of recombination. When a detailed analyses of the number of domestic and peridomestic/sylvatic genotypes was observed among the polymorphic genes (Figure 1). We could determine a high number of genotypes in the peridomestic/sylvatic clones as strong signals of cryptic sexuality but a low number of genotypes among TcI_DOM_ clones. This likely suggest that *T. cruzi* exhibits a high frequency of genetic exchange with a later clonal expansion of specific genotypes that become stable in space and time. Therefore, TcI_DOM_ has become and might be the sibling of multiple recombination events. In terms of parasite success, as the parasite interacts with its host, the parasite becomes less virulent and produces pathologies that are more often associated with long chronic disease; this is the case of TcI_DOM_ that has been related with severe forms of end-stage chronic cardiomyopathy in Colombia and Argentina compared to sylvatic strains which support the premises herein mentioned [49].

In terms of evolutionary trends, we calculated the ratio of nonsynomymous to synonymous changes (Table 1). In these calculations, patterns of positive selection (STTP2, RHO1 and TR) and/or stabilizing selection were detected (GPX, HCOAR, PDH, GTP, SODA, SODB, LAP, GPI, LYT1 and RB19). When these genes were applied to the six DTUs within the *T. cruzi* taxon, it was possible to determine that most genes were under stabilizing selection and just a minority under positive selection [23]. In terms of natural history of TcI the fact of detecting that most of the genes are under stabilizing selection implies that the emergence of genotypes within this DTU is possible and this genotype may be orthodoxically an event of recombination between genes that probably display positive selection favouring the appearance of alleles that increase the frequency of certain trait among the population [50].

## Conclusions

In conclusion, herein we conducted nMLST scheme to unravel the genetic structure in space and time of *T. cruzi* I. Our results demonstrate that TcI exhibits a pattern of unstable genotype among the six Discrete Typing Units of *T. cruzi;* a pattern that may be explained by the diverse number of insect vector and mammals that can be found naturally infected with this DTU. Two robust monophyletic groups were described (TcI_DOM_ and peridomestic/sylvatic cluster) where the status of emergent genotype was described. Many efforts have been conducted to unravel the natural history of this DTU but this robust simplification of the taxonomy should rely on biological and clinical properties of the parasite. We suggest the scientific community to avoid DTU subdivision and to begin using the term of genotype emergence, being TcI_DOM_ the emergent genotype within TcI. We conclude stating the need to pursue studies to elucidate the effect of this specific genotype to the severity of human infection and also the role that recombination plays in the diversification of the parasite and the outcome of Chagas disease in the Americas.

## Methods

### Study area, parasite cloning and T. cruzi genotyping

We selected 50 biological clones belonging to 45 strains. The selection criteria was based on DAS Neighbour Joining tree obtained using STR information where these clones displayed to be the more divergent ones at nuclear level [19]. These isolates were obtained from three different geographical areas (Boyaca, Casanare and Santander) in northeastern Colombia from 2000 to 2011, comprising a geographical sampling area of more than 65.000 Km^2^. The places of sampling were at altitudes that range from 300 to 1800 m.a.s.l. including savanna and mountains, the owners of the lands gave permission to conduct the study on these sites. Triatomines (*Rhodnius prolixus*, *Triatoma dimidiata, T. maculata* and *Eratyrus cuspidatus*) and mammals (*Rattus ratus, Didelphis marsupialis, Tamandua tetradactyla, Alouatta seniculus* and *Proechymis* spp.) were captured at domestic (within dwellings), peridomestic (near dwellings) and sylvatic (more than 10 meters from dwellings). The animals were anesthesized and a blood sample of 1–2 mL was collected, after blood collection the animals were released and manipulated following the international guiding principles for biomedical research involving animals, as issued by the Council for International Organizations of Medical Sciences.

Trypanosomes from Human patients were isolated following all the ethical clearance using written informed consents approved by the Colombian National Health Institute (Additional file 4: Table S1). The parasites isolated were cultured in LIT-biphasic media until they reached exponential phase. The parasites were submitted to single-celled cloning using two methods (FACS and limiting dilution) [51,52]. In total 50 clones were obtained; the clones’ cultures on exponential phase were submitted to DNA extraction taking 200-μL aliquots using a Qiamp DNA isolation Kit. The DNA quality and concentration was measured at 260 nm and stored at -20C. The *T. cruzi* molecular detection was accomplished amplifying the variable region of minicircle kinetoplast DNA using primers 121 and 122 [49]. The genotyping of *T. cruzi* DTUs was developed by direct sequencing of Glucose-Phosphate-Isomerase (GPI) fragment gene region using primers GPI-fwd and GPI-Rv. The sequences obtained were compared with a set of reference strains belonging to the different six DTUs [53].

### Ethics statement

All of the appropriate ethical clearance was considered and the ethics committee from Universidad de los Andes specifically approved this study under the form number 066/2006. Written consent was obtained in all human patients included as part of the epidemiological surveillance developed by NHI and Universidad de Los Andes under the same form number 066/2006.

### Nuclear Multilocus Sequence Typing (nMLST) loci amplification

Fifty TcI single-celled clones were selected as a sample of the total nuclear diversity identified across the 24 STR loci [19]. Thirteen nuclear DNA fragments were selected according to the results of two nMLST schemes previously reported [9,23]. Primers flanking Glutathione peroxidase (GPX), 3-Hydroxy-3-methylglutaryl-CoA reductase (HMCOAR), Pyruvate dehydrogenase component E1 subunit alpha (PDH), Small GTP-binding protein Rab7 (GTP), Serine/threonine-protein phosphatase PP1 (STPP), Rho-like GTP binding protein (RHO1), Glucose-6-Phosphate isomerase (GPI), Superoxide dismutase A (SODA), Superoxide dismutase B (SODB), Leucine aminopeptidase (LAP), Trypanothion reductase (TR), RNA binding-protein 19 (RB19) and Lytic pathway protein (LYT1) were employed (Additional file 5: Table S3). These genes were selected based on the number of genotypes previously described for TcI [9,23]. The amplification was accomplished in a final volume of 20 μL using 1X Buffer (Corpogen, COL), 50 mM MgCl_2_, 10 μM of each primer, 5U/μL of Taq Tucan (Corpogen, COL) and 20 ng of DNA. The mix was submitted to 29 cycles of amplification and the amplicons were visualized in 2% agarose gels stained with gel red.

### Sequence analyzes and phylogenetic reconstructions

The PCR products were cleaned up by isopropanol precipitation and sequenced by the dideoxy-terminal method in an automated capillary sequencer (AB3730, Applied Biosystems, UK) by both strands. The resulting sequences were edited in MEGA 5.0 and aligned using ClustalW 1.8. No ambiguous or heterozygosis positions were found among the sequences. All edited sequences were deposited in GenBank and assigned accession numbers (KF439872-KF440257). The final sequences were concatenated in SeaView 4.0. The final set of concatenated sequences and each individual gene fragment were evaluated in ModelTest 3.7 where the most appropriate evolutionary model was selected based on the AIC (Akaike Information Criterion). A maximum composite likelihood (MCL) analysis using a Tamura-3 parameter model and the Neighbour-Joining algorithm was run in RAxML 7.2.5 on the CIPRES project (*Cyberinfrastructure for Phylogenetic Research*) portal 2.0 servers. Trees were constructed for the concatenated sequences and the individual gene fragments. To evaluate the robustness of the nodes in the resulting phylogenetic trees, 1000 bootstrap replicates were performed. The final trees were rooted with Esmeraldo (TcII) and CANIII (TcIV) strains as reference nMLST DNA sequences.

### Genetic diversity and Diploid Sequence Types (DSTs) analyzes

Sequence genetic diversity was estimated for each gene fragment for the set of 50 TcI clones. π and θ nucleotide diversity indexes and genotype diversity were calculated in DNAsp v.5.0. The number of DSTs for each gene fragment was identified from SNPs across the 50 TcI clones. We also calculated the number of genotypes, variable sites and typing efficiency for each gene fragment. DSTs were subsequently submitted to eBURST software to presume the evolutionary relationships and founders. The eBURST algorithm (http://eburst.mlst.net accessed on September 17 of 2012) identifies related sequences and predicts a founding genotype with variants identified depending on the number of different loci. Likewise, the co-joined DSTs were submitted to LIAN software to obtain a measure of linkage equilibrium. LIAN is a program to test the null hypothesis of linkage equilibrium for multilocus data. LIAN incorporates both a Monte Carlo method as well as a novel algebraic method to carry out the hypothesis test. The program further returns the genetic diversity of the sample and the pairwise distances between its members [54]. Additionally, the ratio of non-synonymous to synonymous amino acid changes (dN/dS) was calculated according to Nei-Gojobori method using SNAP software (http://www.hiv.lanl.gov accessed on September 17 of 2012) to determine plausible selection pressures.

We constructed NJ trees based on the DSTs profiles that were compared to phylogenetic trees to test the robustness of the multilocus data. In order to observe plausible events of recombination, the whole dataset of concatenated sequences was submitted to analysis in RDP v.3.0 searching recombination breaking points using three approaches; GENECONV finds the most likely candidates for aligned gene conversion events between pairs of sequences in the alignment. Candidate events are ranked by multiple-comparison corrected p-values and listed to a spreadsheet-like output file, BOOTSCAN/RECSCAN screens nucleotide sequence alignments for evidence of recombination without prior identification of non-recombinant reference sequences, this algorithm includes a Bonferroni corrected statistical test of recombination and MAXCHI is based on *X*^2^ statistical method and creates contingency tables according to the probability of finding recombination events in the alignment analyzed.

## Competing interests

The authors declare that no conflict or competing interests exist regarding this manuscript.

## Authors’ contributions

JDR and GT performed the experiments, JDR designed the experiments, JDR and FG analyzed the data, JDR and FG wrote the paper. All authors read and approved the final manuscript.

## Supplementary Material

Additional file 1: Figure S1 Phylogeny incongruence between nuclear markers applied to the 50 biological TcI clones analyzed. A. ML phylogenetic reconstruction using the SNP’s from HMCOAR gene B. ML phylogenetic reconstruction using the SNP’s from SODB gene. Click here for file

Additional file 2: Table S2 Diploid sequence profiles and Diploid Sequence Types (DSTs) for the 50 TcI biological clones studied by the 13 nuclear MLST scheme markers. Click here for file

Additional file 3: Figure S2 Output of RDP software showing the recombination breaking points of two clones A. CACQcl7 B. NA2cl4. Click here for file

Additional file 4: Table S1 Biological and geographical distribution of *Trypanosoma cruzi* I biological clones isolated from humans, triatomine bugs and reservoirs from three endemic areas of Chagas disease in Colombia (Boyacá, Casanare and Santander) submitted to nMLST analysis. Click here for file

Additional file 5: Table S3 Details of Gene targets employed in the nMLST on the 50 TcI clones. Click here for file
