# Supplementary material for: Genetic structure of Trypanosoma cruzi in Colombia revealed by a High-throughput Nuclear Multilocus Sequence Typing (nMLST) approach
Source: BMC Genet. 2013 Sep 30;14:96. doi: 10.1186/1471-2156-14-96 (PMC3850472; doi:10.1186/1471-2156-14-96)
Supplement: Additional file 2: Table S2 — Diploid sequence profiles and Diploid Sequence Types (DSTs) for the 50 TcI biological clones studied by the 13 nuclear MLST scheme markers. [file 1471-2156-14-96-S2.docx]

**Table S2.** Diploid sequence profiles and Diploid Sequence Types (DST´s) for the 50 TcI biological clones studied by the 13 nuclear MLST scheme markers.

| **Clone code** | **GPX** | **HMCOAR** | **PDH** | **GTP** | **STPP2** | **RHO1** | **SODA** | **SODB** | **LAP** | **GPI** | **LYT1** | **RB19** | **TR** | **DST** |
| --- | --- | --- | --- | --- | --- | --- | --- | --- | --- | --- | --- | --- | --- | --- |
| AAC1cl3 | 1 | 1 | 1 | 1 | 1 | 1 | 1 | 1 | 1 | 1 | 1 | 1 | 1 | 1 |
| AAD6cl6 | 2 | 2 | 2 | 1 | 1 | 1 | 1 | 2 | 1 | 2 | 2 | 1 | 1 | 2 |
| AADm1cl2 | 3 | 3 | 1 | 1 | 1 | 1 | 1 | 3 | 1 | 3 | 3 | 1 | 1 | 3 |
| CACQcl7 | 4 | 4 | 3 | 2 | 2 | 2 | 2 | 4 | 2 | 4 | 4 | 2 | 2 | 4 |
| Cepa2cl8 | 5 | 5 | 1 | 3 | 1 | 3 | 1 | 5 | 3 | 5 | 5 | 1 | 1 | 5 |
| DAcl15 | 6 | 6 | 4 | 4 | 3 | 4 | 3 | 6 | 4 | 6 | 6 | 3 | 3 | 6 |
| DYRcl5 | 6 | 6 | 4 | 4 | 2 | 4 | 4 | 6 | 4 | 6 | 6 | 4 | 2 | 7 |
| EBcl12 | 7 | 6 | 4 | 5 | 2 | 5 | 3 | 7 | 5 | 6 | 7 | 3 | 2 | 8 |
| EHcl5 | 8 | 6 | 5 | 5 | 2 | 6 | 5 | 8 | 6 | 6 | 8 | 5 | 2 | 9 |
| EMcl4 | 6 | 6 | 6 | 4 | 3 | 7 | 3 | 9 | 7 | 6 | 6 | 3 | 3 | 10 |
| FECcl10 | 6 | 7 | 5 | 5 | 2 | 7 | 6 | 6 | 7 | 7 | 6 | 6 | 2 | 11 |
| H10cl10 | 9 | 8 | 7 | 6 | 4 | 8 | 7 | 10 | 8 | 8 | 9 | 7 | 4 | 12 |
| LCVcl11 | 10 | 6 | 4 | 4 | 2 | 7 | 5 | 4 | 7 | 6 | 10 | 5 | 2 | 13 |
| LERcl11 | 11 | 6 | 5 | 5 | 2 | 9 | 5 | 11 | 9 | 6 | 11 | 5 | 2 | 14 |
| LERcl14 | 12 | 6 | 5 | 4 | 2 | 10 | 5 | 7 | 10 | 6 | 12 | 5 | 2 | 15 |
| LERcl15 | 4 | 6 | 8 | 7 | 2 | 6 | 5 | 8 | 6 | 6 | 4 | 5 | 2 | 16 |
| LJVPcl7 | 13 | 6 | 4 | 8 | 5 | 10 | 4 | 8 | 10 | 6 | 13 | 4 | 5 | 17 |
| LNcl1 | 8 | 7 | 5 | 9 | 5 | 11 | 8 | 9 | 11 | 7 | 8 | 8 | 5 | 18 |
| N5P14cl3 | 14 | 9 | 9 | 10 | 1 | 12 | 9 | 5 | 12 | 9 | 14 | 9 | 1 | 19 |
| NA2cl4 | 15 | 9 | 10 | 10 | 1 | 13 | 10 | 12 | 13 | 9 | 15 | 10 | 1 | 20 |
| NB2cl4 | 15 | 5 | 11 | 10 | 1 | 13 | 7 | 12 | 13 | 5 | 15 | 7 | 1 | 21 |
| NC2cl8 | 15 | 1 | 11 | 10 | 1 | 13 | 9 | 12 | 13 | 1 | 15 | 9 | 1 | 22 |
| NR1cl3 | 15 | 1 | 11 | 11 | 1 | 14 | 7 | 12 | 14 | 1 | 15 | 7 | 1 | 23 |
| RGRcl10 | 16 | 5 | 12 | 12 | 6 | 15 | 11 | 13 | 15 | 5 | 16 | 11 | 6 | 24 |
| SEVcl21 | 17 | 7 | 13 | 13 | 2 | 16 | 4 | 8 | 16 | 7 | 17 | 4 | 2 | 25 |
| SLB3cl3 | 14 | 2 | 11 | 10 | 1 | 13 | 7 | 14 | 13 | 2 | 14 | 7 | 1 | 26 |
| SLD2cl3 | 18 | 5 | 14 | 14 | 7 | 17 | 12 | 14 | 17 | 5 | 18 | 12 | 7 | 27 |
| SLDEccl6 | 2 | 5 | 11 | 10 | 8 | 13 | 13 | 15 | 13 | 5 | 2 | 13 | 8 | 28 |
| SLDm1cl7 | 19 | 3 | 11 | 15 | 1 | 18 | 1 | 16 | 18 | 3 | 19 | 1 | 1 | 29 |
| SLDm1cl8 | 20 | 3 | 10 | 15 | 1 | 18 | 1 | 17 | 18 | 3 | 20 | 1 | 1 | 30 |
| SLFcl3 | 21 | 5 | 15 | 15 | 1 | 19 | 7 | 14 | 19 | 5 | 21 | 7 | 1 | 31 |
| SMAcl3 | 22 | 6 | 4 | 16 | 5 | 2 | 14 | 8 | 2 | 6 | 22 | 14 | 5 | 32 |
| SMAcl7 | 22 | 6 | 4 | 17 | 5 | 2 | 14 | 8 | 2 | 6 | 22 | 14 | 5 | 33 |
| SPcl15 | 8 | 7 | 16 | 5 | 2 | 4 | 3 | 6 | 4 | 7 | 8 | 3 | 2 | 34 |
| SR2cl10 | 23 | 9 | 17 | 10 | 1 | 20 | 9 | 17 | 20 | 9 | 23 | 9 | 1 | 35 |
| Td11cl6 | 24 | 8 | 18 | 6 | 4 | 21 | 12 | 10 | 21 | 8 | 24 | 12 | 4 | 36 |
| Td3cl11 | 24 | 8 | 19 | 6 | 6 | 21 | 15 | 10 | 21 | 8 | 24 | 15 | 6 | 37 |
| TmPA1cl6 | 25 | 10 | 20 | 18 | 7 | 22 | 16 | 12 | 22 | 10 | 25 | 16 | 7 | 38 |
| X150cl8 | 26 | 11 | 21 | 19 | 4 | 23 | 12 | 18 | 23 | 11 | 26 | 12 | 4 | 39 |
| X1544cl4 | 24 | 11 | 22 | 19 | 4 | 23 | 12 | 18 | 23 | 11 | 24 | 12 | 4 | 40 |
| X236cl9 | 24 | 8 | 23 | 19 | 9 | 23 | 12 | 18 | 23 | 8 | 24 | 12 | 9 | 41 |
| X380cl20 | 26 | 11 | 24 | 19 | 6 | 23 | 12 | 18 | 23 | 11 | 26 | 12 | 6 | 42 |
| XcHcl15 | 8 | 7 | 25 | 9 | 5 | 11 | 8 | 9 | 11 | 7 | 8 | 8 | 5 | 43 |
| YAScl3 | 25 | 12 | 26 | 20 | 10 | 24 | 9 | 12 | 24 | 12 | 25 | 9 | 10 | 44 |
| YB1cl2 | 27 | 5 | 27 | 15 | 6 | 25 | 17 | 17 | 25 | 5 | 27 | 17 | 6 | 45 |
| YDm1Bcl4 | 28 | 12 | 1 | 21 | 11 | 18 | 18 | 17 | 18 | 12 | 28 | 18 | 11 | 46 |
| YDm1Mcl4 | 28 | 3 | 28 | 21 | 11 | 18 | 18 | 12 | 18 | 3 | 28 | 18 | 11 | 47 |
| YTT1cl2 | 25 | 12 | 28 | 22 | 6 | 24 | 12 | 17 | 24 | 12 | 25 | 12 | 6 | 48 |
| YASCl2 | 25 | 12 | 28 | 22 | 6 | 24 | 12 | 17 | 24 | 12 | 25 | 12 | 6 | 49 |
| YTT1cl3 | 27 | 5 | 27 | 15 | 6 | 25 | 17 | 17 | 25 | 5 | 27 | 17 | 6 | 50 |
