# Supplementary figures and images for: Genetic structure of Trypanosoma cruzi in Colombia revealed by a High-throughput Nuclear Multilocus Sequence Typing (nMLST) approach
Source: BMC Genet. 2013 Sep 30;14:96. doi: 10.1186/1471-2156-14-96 (PMC3850472; doi:10.1186/1471-2156-14-96)

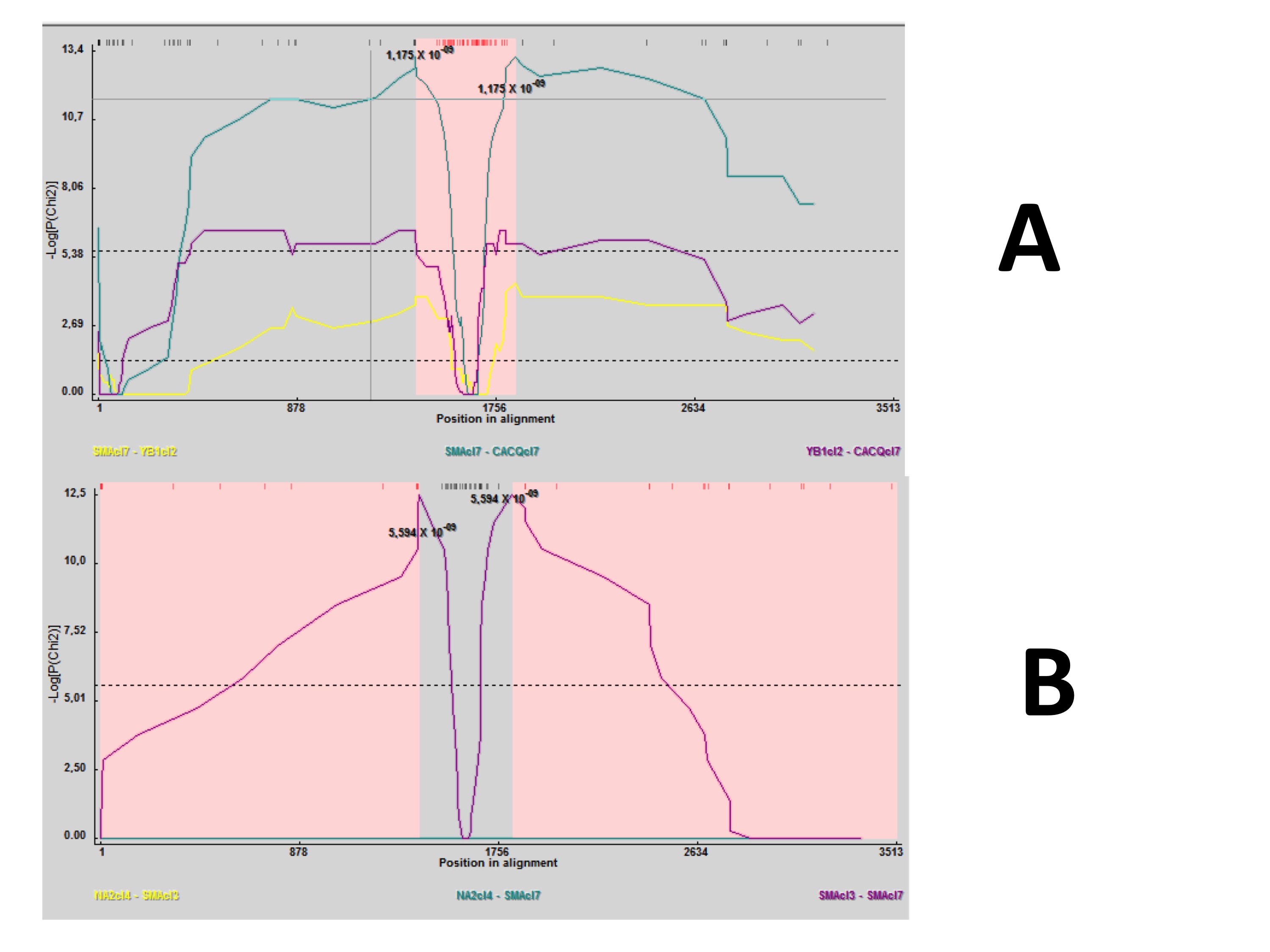

Supplement: Additional file 3: Figure S2 — Output of RDP software showing the recombination breaking points of two clones A. CACQcl7 B. NA2cl4. [file 1471-2156-14-96-S3.jpeg]
