# Supplementary material for: Genetic structure of Trypanosoma cruzi in Colombia revealed by a High-throughput Nuclear Multilocus Sequence Typing (nMLST) approach
Source: BMC Genet. 2013 Sep 30;14:96. doi: 10.1186/1471-2156-14-96 (PMC3850472; doi:10.1186/1471-2156-14-96)
Supplement: Additional file 4: Table S1 — Biological and geographical distribution of Trypanosoma cruzi I biological clones isolated from humans, triatomine bugs and reservoirs from three endemic areas of Chagas disease in Colombia (Boyacá, Casanare and Santander) submitted to nMLST analysis. [file 1471-2156-14-96-S4.docx]

**Table S1.** Biological and geographical distribution of *Trypanosoma cruzi* I biological clones isolated from humans, triatomine bugs and reservoirs from three endemic areas of Chagas disease in Colombia (Boyacá, Casanare and Santander) submitted to nMLST analysis.

| **Clone code** | **Host** | **Geographical area** | **Genotype** | **Transmission cycle** |
| --- | --- | --- | --- | --- |
| AAC1cl3 | *Rhodnius prolixus* | Casanare | TcI | Sylvatic |
| AAD6cl6 | *Rhodnius prolixus* | Casanare | TcI | Sylvatic |
| AADm1cl2 | *Didelphis marsupialis* | Casanare | TcI | Sylvatic |
| CACQcl7 | *Homo sapiens* | Santander | TcI | Domestic |
| Cepa2cl8 | *Rodent* | Casanare | TcI | Sylvatic |
| DAcl15 | *Homo sapiens* | Boyacá | TcI | Domestic |
| DYRcl5 | *Homo sapiens* | Boyacá | TcI | Domestic |
| EBcl12 | *Homo sapiens* | Boyacá | TcI | Domestic |
| EHcl5 | *Homo sapiens* | Boyacá | TcI | Domestic |
| EMcl4 | *Homo sapiens* | Boyacá | TcI | Domestic |
| FECcl10 | *Homo sapiens* | Boyacá | TcI | Domestic |
| H10cl10 | *Canis familiaris* | Boyacá | TcI | Sylvatic |
| LCVcl11 | *Homo sapiens* | Santander | TcI | Domestic |
| LERcl11 | *Homo sapiens* | Santander | TcI | Domestic |
| LERcl14 | *Homo sapiens* | Santander | TcI | Domestic |
| LERcl15 | *Homo sapiens* | Santander | TcI | Domestic |
| LJVPcl7 | *Homo sapiens* | Santander | TcI | Domestic |
| LNcl1 | *Homo sapiens* | Santander | TcI | Domestic |
| N5P14cl3 | *Rhodnius prolixus* | Casanare | TcI | Sylvatic |
| NA2cl4 | *Rhodnius prolixus* | Casanare | TcI | Sylvatic |
| NB2cl4 | *Rhodnius prolixus* | Casanare | TcI | Sylvatic |
| NC2cl8 | *Rhodnius prolixus* | Casanare | TcI | Sylvatic |
| NR1cl3 | *Rattus rattus* | Casanare | TcI | Sylvatic |
| RGRcl10 | *Rhodnius prolixus* | Casanare | TcI | Sylvatic |
| SEVcl21 | *Homo sapiens* | Boyacá | TcI | Domestic |
| SLB3cl3 | *Rhodnius prolixus* | Casanare | TcI | Sylvatic |
| SLD2cl3 | *Rhodnius prolixus* | Casanare | TcI | Sylvatic |
| SLDEccl6 | *Eratyrus cuspidatus* | Casanare | TcI | Sylvatic |
| SLDm1cl7 | *Didelphis marsupialis* | Casanare | TcI | Sylvatic |
| SLDm1cl8 | *Didelphis marsupialis* | Casanare | TcI | Sylvatic |
| SLFcl3 | *Rhodnius prolixus* | Casanare | TcI | Sylvatic |
| SMAcl3 | *Homo sapiens* | Santander | TcI | Domestic |
| SMAcl7 | *Homo sapiens* | Santander | TcI | Domestic |
| SPcl15 | *Homo sapiens* | Casanare | TcI | Domestic |
| SR2cl10 | *Rodent* | Casanare | TcI | Sylvatic |
| Td11cl6 | *Triatoma dimidiata* | Boyacá | TcI | Sylvatic |
| Td3cl11 | *Triatoma dimidiata* | Boyacá | TcI | Sylvatic |
| TmPA1cl6 | *Triatoma maculata* | Casanare | TcI | Sylvatic |
| X150cl8 | *Rhodnius prolixus* | Boyacá | TcI | Sylvatic |
| X1544cl4 | *Rhodnius prolixus* | Boyacá | TcI | Sylvatic |
| X236cl9 | *Rhodnius prolixus* | Boyacá | TcI | Sylvatic |
| X380cl20 | *Rhodnius prolixus* | Boyacá | TcI | Sylvatic |
| XcHcl15 | *Homo sapiens* | Boyacá | TcI | Domestic |
| YAScl3 | *Alouatta seniculus* | Casanare | TcI | Sylvatic |
| YB1cl2 | *Rhodnius prolixus* | Casanare | TcI | Sylvatic |
| YDm1Bcl4 | *Didelphis marsupialis* | Casanare | TcI | Sylvatic |
| YDm1Mcl4 | *Didelphis marsupialis* | Casanare | TcI | Sylvatic |
| YTT1cl2 | *Tamandua tetradactyla* | Casanare | TcI | Sylvatic |
| YASCl2 | *Alouatta seniculus* | Casanare | TcI | Sylvatic |
| YTT1cl3 | *Tamandua tetradactyla* | Casanare | TcI | Sylvatic |
