# Supplementary material for: Genetic structure of Trypanosoma cruzi in Colombia revealed by a High-throughput Nuclear Multilocus Sequence Typing (nMLST) approach
Source: BMC Genet. 2013 Sep 30;14:96. doi: 10.1186/1471-2156-14-96 (PMC3850472; doi:10.1186/1471-2156-14-96)
Supplement: Additional file 5: Table S3 — Details of Gene targets employed in the nMLST on the 50 TcI clones. [file 1471-2156-14-96-S5.doc]

**Table S3**.Details of Gene targets employed in the nMLST on the 50 TcI clones

| **Gene** | **Length fragment** | **Chromosome location** | **Gene ID** |
| --- | --- | --- | --- |
| GPX | 360bp | 35 | Tc00.1047053511543.60 |
| HMCOAR | 554bp | 32 | Tc00.1047053506831.40 |
| PDH | 491bp | 40 | Tc00.1047053507831.70 |
| GTP | 561bp | 12 | Tc00.1047053503689.10 |
| STTP2 | 409bp | 34 | Tc00.1047053507673.10 |
| RHO1 | 455bp | 8 | Tc00.1047053506649.40 |
| GPI | 405bp | 6 | Tc00.1047053506529.508 |
| SODA | 300bp | 21 | Tc00.1047053509775.40 |
| SODB | 335bp | 35 | Tc00.1047053507039.10 |
| LAP | 420bp | 27 | Tc00.1047053508799.240 |
| TR | 602bp | 37 | Tc00.1047053503555.30 |
| RB19 | 350bp | 29 | Tc00.1047053507515.60 |
| LYT1 | 691bp | 22 | Tc00.1047053508045.40 |
